# Of the Drug Curative

**Published:** 1890-01

**Authors:** P. P. Wells


### OF THE DRUG CURATIVE.

Dr. P. P. Wells.

Communicated by C. Carleton Smith, M. D., Philadelphia.

It is difficult not to sympathize with those who declare the absurdity of the highest potencies, if the power to cure be only material in its nature. We cannot conceive of *matter* being continually expanded through all these successive numbers, in a centesimal ratio, and yet, in the 40,000th number of the series retain a sufficient quantity of the matter of the drug to cure so important a disease as Asiatic cholera. And yet, this actually occurred in a case seen by the writer in consultation, which he knows positively was no other than this most formidable disease.

In the case there was no symptom wanting to establish its true character beyond a doubt. Veratrum was given, in ordinary numbers, high and low, for there was no question as to the simillimum for the case. The relief which followed each dose was partial and temporary, but in spite of all, the patient rather lost than gained, till the attendant in a happy moment determined to give his patient a dose of Fincke's 40,000th of this drug. The result was an immediate and complete success. The convalescence was rapid, and in all respects satisfactory, though the patient, a female, was sixty years of age. This certainly shows the highest use. If there had been no other cure from these high potencies, this one is enough to raise their author far above the reproaches which have been cast upon him on their account. But there have been many cures from these when other preparations of the same drug have failed. We repeat, these many instances of cures from these potencies, where others, and lower, had failed, testify to the true nature of the curative principle, in the demonstration they present of a greater power to cure with a less quantity of matter; thus showing conclusively that the power that cures is *not matter*. To this conclusion we are compelled, unless we adopt the absurdity of the less exceeding the greater. These cures are many, and they are *facts*. For those who have no knowledge of the individual cases, to deny the facts they set forth, for no better reason than their own skepticism, is simply an impertinence, not deserving the least respect. We only add, that in this, the curative principle exhibits an entire disregard of the laws of matter, and obeys only its own, which are *laws of force*.

From these facts it seems evident that the curative principle may be, and has been, separated from the orginal material association, and made free to follow the laws of its own independent existence. It is no longer to be judged of as to its own habitudes, in the light of what is supposed to be known of the material bodies with which it was previously associated. Not being itself matter a true knowledge of it can be extended only by studying the principle in its new conditions and relations. Of this we may have more to say in the future. We only add to the fact here, as applicable to the next example we are about to introduce of its disregard of material laws, which we think goes far of itself to establish the truth advocated in this paper. Taken in connection with the other known facts of its operations and relations to other bodies and forces, the truth seems to be placed by them beyond dispute. The fact to which we allude has received from one of its distinguished observers the nomination of "*potentization by contact.*" By this is meant to express in brief terms the fact that bodies charged with this curative principle, set free from its material drug association, impart it to neutral bodies, when brought into close contact with them, so that these last become medicated by this contact, and are made capable of affecting the living organism equally with other bodies similarly charged. By this contact they receive all the powers, and enter into all the curative relations of the original charged body. In other words, medicated globules brought into contact with those which are not medicated, and being so kept for a time, impart the medicinal or curative power they possess to the whole mass of neutrals.

That this actually occurs has been testified to by many of the best observers. They have often *proved* the fact by experiment, and therefore their testimony must stand against whatever of denial or sneer, from those who have made no such experiment.

Of those who have proved and asserted this truth, we name only one, and he, so far as we know, was the first to observe it. This was Dr. Wm. C. Channing, late of New York City. To those who, like the writer, had the privilege of acquaintance with Dr. C. it is only necessary to mention his name, and he stands before them in memory as he was in life, a man of noble intellect, of rare attainments in secular and professional scholarship, with talents for observation surpassed by none, and an integrity never doubted by any, however they may have differed from him in opinions. Dr. C. first called the attention of the writer to the fact of potentization by contact, by relating what had then been his experience in the matter for a number of years.

He had carefully observed, noted and practiced on it long after he had established the truth of the phenomenon. It was of his experience with *Aconite* that he spoke. He had, at that time been carrying a phial in his pocket-case for a number of years, which, in the beginning, was filled with globules charged with the 30th centesimal potency of this drug, then the highest number in use. Many times, and whenever the phial was nearly empty of its charged globules, he added others, not medicated, shook the whole contents till they were well mixed, and then, after a short time, he used from the phial as at the first, and with the same practical success as before.

This, as we have said, had then been many times repeated, and at last as at the first, the same curative results followed the use of these globules so medicated, in this hitherto unheard-of way, as he had before witnessed some globules charged in the usual way. At that time Aconite was probably more frequently used in practice than at the present. In those of Dr. C. it continued to be as often a reliable curative as in the hands of any other man. In his practice Dr. C. was eminently successful as is well known by many of his surviving colleagues.

Now the globules as used by Dr. C. were medicated by contact or were not medicated at all. In the latter case, he used blank globules for years supposing them to be medicated, deceiving himself, his patients (who were satisfactorily cured) and the public. He never once realized the specific effects of *Aconite*, though he thought he saw them constantly. His patients were cured by *nothing*, though apparently they were cured by medication. He and they were thus so grossly deceived that they never questioned the genuineness of the cures. If, as we have no reason to doubt, they were genuine, they are each of them witnesses to the truth of *medication* by *contact*.

